# NK Cells Can Preferentially Target Prostate Cancer Stem-like Cells via the TRAIL/DR5 Signaling Pathway

**DOI:** 10.3390/biom11111702

**Published:** 2021-11-16

**Authors:** Taiga Seki, Yui Shimizu, Kyota Ishii, Yuzuki Takahama, Kazunori Kato, Tomohiro Yano

**Affiliations:** 1 Graduate School of Food and Nutritional Sciences, Toyo University, Gunma 347-0193, Japan; s3c102000049@toyo.jp (T.S.); s3c102100017@toyo.jp (K.I.); s3c102000050@toyo.jp (Y.T.); 2 Graduate School of Science and Engineering, Toyo University, Saitama 350-8555, Japan; yui.080720@gmail.com (Y.S.); k-kato@toyo.jp (K.K.); 3 Research Institute of Life Innovation, Toyo University, Gunma 347-0193, Japan

**Keywords:** androgen-dependent prostate cancer, stem cells, NK cells, cytotoxicity, TRAIL/DR5 signal pathway

## Abstract

Background: The occurrence of androgen-dependent prostate cancer mainly depends on prostate cancer stem cells. To reduce the risk of androgen-dependent prostate cancer, the direct elimination of prostate cancer stem cells is important, but an elimination strategy has not yet been established. A previous study showed that natural killer (NK) cells can preferentially target cancer stem cells in several solid tumors except prostate cancer. In this context, this study was undertaken to investigate if NK cells can selectively attack androgen-dependent prostate cancer stem cells. Methods: Prostate cancer stem-like cells were separated from an androgen-dependent prostate cancer cell line (LNCaP) using a three-dimensional culture system. LNCaP stem-like cells or LNCaP cells were co-cultured with human NK cells (KHYG-1) for 24–72 h, and cell viability was determined using the WST-8 method. The expression of each protein in the cell membrane was evaluated through FACS analysis, and mRNA levels were determined using real-time PCR. Results: KHYG-1 cells had more potent cytotoxicity against LNCaP stem-like cells than LNCaP cells, and the potency of the cytotoxicity was strongly related to the TRAIL/DR5 cell death pathway. Conclusion: NK cells can preferentially target prostate cancer stem-like cells via the TRAIL/DR5 pathway.

## 1. Introduction

Among male-specific diseases, prostate cancer represents a major health risk worldwide. Cancer statistics for the USA in 2018 estimated the number of cases and subsequent deaths from prostate cancer to be 164,900 and 29,430, respectively [1]. During the early stages of disease, prostate cancer cells grow and survive in an androgen-dependent manner, so androgen deprivation therapy is initially effective [2]. However, the efficacy of the therapy is short, and, ultimately, prostate cancer cells become androgen-independent and have several malignant phenotypes, such as castration-resistant prostate cancer (CRPC) [3]. Since androgen-independent prostate cancers remain incurable because of their resistance to androgen deprivation therapy [4], an effective preventive approach that can suppress the appearance of CPRC is urgently required to improve prostate cancer survival rates.

Cancer stem-like cells are minor and undifferentiated cell populations in cancer tissues and have been proposed to be the primary mediators of tumor initiation, progression, recurrence, metastasis, and resistance to treatment [5,6]. Moreover, prostate cancer stem-like cells may be responsible for the occurrence and recurrence of prostate cancer and the appearance of the androgen-independent and incurable phenotype [7,8]. To reduce the occurrence of androgen-dependent prostate cancer and the development of the androgen-independent phenotype after androgen deprivation therapy, the complete and effective eradication of androgen-dependent prostate cancer stem-like cells is required.

Natural killer (NK) cells are a subset of lymphocytes that play a central role in the innate immune response to tumors and infections. They have the ability to kill virally infected or transformed cells via the directed release of lytic granules or by inducing death receptor-mediated apoptosis via the expression of Fas ligand or tumor necrosis factor-related apoptosis-inducing ligand (TRAIL) [9]. Furthermore, NK cell-mediated cytotoxic activity is closely associated with the occurrence of cancer, and decreased NK cell activity contributes to an increased risk of cancer [10]. As mentioned, the complete eradication of androgen-dependent prostate cancer stem-like cells could lead to an effective reduction in the risk of prostate cancer occurrence. Taken together, it seems possible that activated NK cells could reduce the occurrence risk of prostate cancer via the effective elimination of androgen-dependent prostate cancer stem-like cells. In fact, it has been reported that NK cells preferentially induce cytotoxicity against cancer stem cells within a non-cancer stem cell population [11]. In this context, the present study was undertaken to clarify if NK cells can preferentially eradicate prostate cancer stem-like cells and, if so, to determine which pathways contribute to NK cell-mediated cytotoxicity against cancer stem-like cells.

## 2. Materials and Methods

### 2.1. Reagents

All reagents were purchased from Nakarai Tesque (Kyoto, Japan), Wako Chemicals (Osaka, Japan), and Pepro Tech (Cranbury, NJ, USA), unless otherwise indicated. Fetal bovine serum (FBS) was purchased from Bio West (Nuaillé, France). Phycoerythrin (PE)-conjugated antibodies for flow cytometry (FACS) were PE-IgG1 isotype Ctrl, PE-anti-human CD262 (DR5, TRAIL-R2), PE-anti-human CD261 (DR4, TRAIL-R1), PE-anti-human DR3 (TRAMP), PE-anti-human DcR1 (TRAIL-R3, CD263), PE-anti-human DcR2 (CCR6), PE-anti-human MICA/MICB, PE-anti-human CD335 (NKp46), and PE-anti-human CD337 (NKp30) from Bio Legend (San Diego, CA, USA); PE-anti-hULBP-1 and PE-anti-hULBP-2/5/6 from R & D Systems (Minneapolis, MN, USA); and PE-anti-human NKG2D (activating) from eBioscience (San Diego, CA, USA). PerCP/cy5.5-conjugated antibodies for FACS were PerCP/cy5.5 Mouse IgG1 isotype Ctrl, PerCP/cy5.5 anti-human CD56 (NCAM), PerCP/cy5.5 anti-human CD96 (TACTILE), and PerCP/Cyanine5.5 anti-human CD3 from Bio Legend. PCR primers were obtained from Nihon Gene Research Laboratories (Miyagi, Japan).

### 2.2. Cell Culture and Preparation of Prostate Cancer Stem-like Cells

LNCaP cells (American Type Culture Collection, Manassas, VA, USA) were used as a typical human androgen-dependent prostate cancer cell line [12], and KHYG-1 cells (JCRB Cell Bank, Osaka, Japan), which have a similar phenotype to human NK cells [13], were utilized as a representative NK cell line. The cells were routinely grown in RPMI-1640 supplemented with 10% FBS, 100 U/mL penicillin, and 100 μg/mL streptomycin at 37 °C in a humidified atmosphere with 5% CO_2_. To stimulate the growth of KHYG-1 cells, recombinant human IL-2 (50 ng/mL) was added to the culture medium. To isolate LNCaP stem-like (LN-stem) cells from LNCaP parental (LN) cells, we utilized the tumorsphere-forming capacity of the cells in a three-dimensional (3D) culture system using a low attachment culture plate (Sumitomo Bakelite Co., Ltd., Tokyo, Japan) [14]. In the study, we checked the mRNA and protein levels of some cancer stem cell markers (CD24, CD44, CD133, SOX2, Oct3/4) and the tumorsphere-forming capacity of the 3D culture to confirm stemness of the cells from tumorsphere [14]. Moreover, every time we prepared the cells from tumorspheres in this study, we checked the mRNA levels of some cancer stem cell markers to validate the stemness of the cells. As the spheroid-forming capacity of LN-stem cells in a two-dimensional (2D) culture using 2% FBS DMEM/F12 medium was retained for four passages, the cells from four passages were used. In the co-culture experiment, LN cells or LN-stem cells were cultured in the 2D culture system for 24 h before culturing with KHYG-1 cells.

### 2.3. Cell Viability Assay

To evaluate the effects of KHYG-1 cells and recombinant human (rh) TRAIL on the viability of LN cells and LN-stem cells, a WST-8 assay was carried out. LN cells and LN-stem cells were seeded in a 96-well plate (5 × 10^3^ cells/well), cultured for 24 h, and subsequently co-cultured with KHYG-1 cells or treated with rf TRAIL for 24–72 h (cell numbers and doses are indicated in their respective figure legends). After each treatment, 10 μL of WST-8 solution was applied to each well containing 100 μL of cell suspension and incubated for a further 30 min at 37 °C in 5% CO_2_. Color development was monitored at 450 nm using a multi-well plate reader (SUNRISE Rainbow RC-R, Tecan Japan, Kanagawa, Japan). For the co-culture experiment, the KHYG-1 cell-containing supernatant was removed after culturing, the well was washed with phosphate-buffered saline (PBS) twice, and the WST-8 solution was added.

### 2.4. Crystal Violet Staining

To further confirm the difference between the KHYG-1 cell-induced cytotoxicity on LN cells and LN-stem cells, crystal violet (CV) staining was carried out. LN cells and LN-stem cells were seeded in a 24-well plate (5 × 10^4^ cells/well), cultured for 24 h, and subsequently co-cultured with KHYG-1 cells for 24 h. After the co-culture, the culture supernatant was discarded, and 300 μL of 4% paraformaldehyde solution was applied to each well and fixed for 15 min. After the fixed cells were washed three times with phosphate-buffered saline, 300 μL of 4% CV solution was added to each well. CV staining was carried out at 37 °C in a humidified atmosphere with 5% CO_2_ for 12 h, and photos were taken of the cells stained with CV at 20× magnification.

### 2.5. Isolation of mRNA and Real-Time Quantitative PCR (qPCR)

Total RNA was isolated from the cells using the Tissue Total RNA Extraction Mini Kit (Favorgen Biotech Corp., Ping-Tung, Taiwan). Total RNA (300 ng for each sample) was used for cDNA synthesis using the ReverTra Ace qPCR RT Kit (Toyobo, Osaka, Japan). cDNA templates were analyzed by real-time PCR using the Thermal Cycler Dice Real Time System Lite (TAKARA BIO INC., Shiga, Japan) and THUNDER-BIRD™ SYBR qPCR Mix (Toyobo, Osaka, Japan), with the following program: 10 s at 95 °C followed by 40 cycles of 15 s at 95 °C and 1 min at 60 °C. Primer sets are shown in Table 1. Gene expression data were normalized to the expression of the reference gene ribosomal protein L32 (RPL32).

### 2.6. Flow Cytometry Analysis

The following antibodies were used: anti-CD3-PerCP-Cy5.5, anti-CD56-PerCP-Cy5.5, anti-CD96-PerCP-Cy5.5, anti-NKG2D-PE, anti-NKp30-PE, anti-NKp46-PE, anti-MICA/B-PE, anti-ULBP-1-PE, anti-ULBP-2/5/6-PE, anti-DcR1-PE, anti-DcR2-PE, anti-DR3-PE, anti-DR4-PE, and anti-DR5-PE. Cells (1 × 10^5^ cells/mL) were incubated with the indicated antibodies for 60 min on ice and washed twice with PBS with 2% FCS. Flow cytometry analysis was performed using FACS Calibur (BD Immunocytometry Systems, Franklin Lakes, NJ, USA), and data were analyzed by Cell Quest software.

### 2.7. Statistical Analysis

Differences among groups were analyzed by one-way ANOVA followed by the Tukey–Kramer test, and differences between two groups were analyzed by one-way ANOVA followed by the Student’s *t*-test. All statistical analyses were performed using Ekuseru-Toukei software (Social Survey Research Information Co., Ltd., Tokyo, Japan). Differences with *p*-values of 0.05 or less were considered statistically significant. All experiments were conducted with a minimum of three samples from three independent experiments, and the data are expressed as the means ± SEM. The number of samples for each experiment is shown in the respective figure legends.

## 3. Results

### 3.1. Comparison between the Sensitivity of LN-Stem Cells and LN Cells to NK Cell-Mediated Cytotoxicity

**Figure 1:**
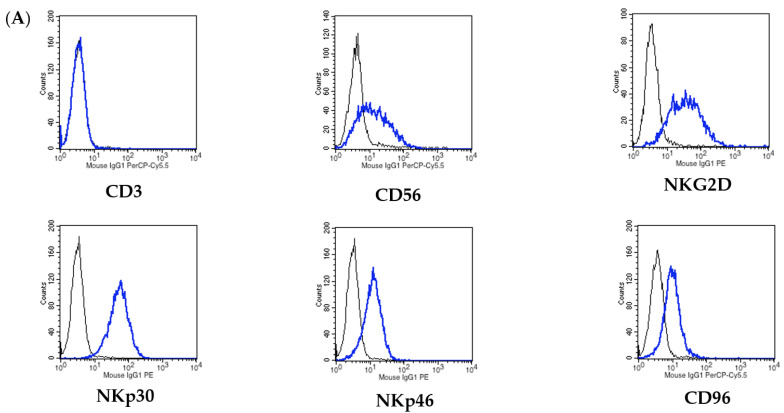

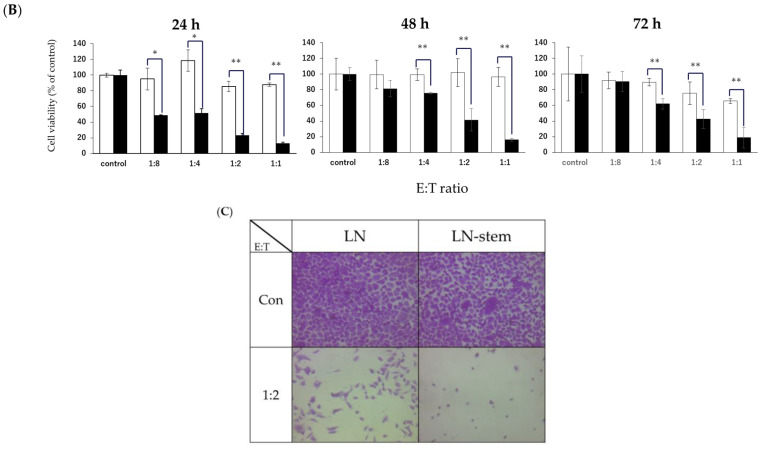
KHYG-1 cells preferentially target LN-stem cells over LN cells. (**A**) Analysis of the expression of each activation receptor on the surfaces of KHYG-1 cells by FACS compared with a matched isotype control (black line). (**B**) Co-culture of KHYG-1 cells with LN-stem cells (filled column) or LN cells (open column) was performed for 24, 48, and 72 h, and cell viability was determined by the WST-8 assay. Cell viability of LN-stem cells or LN cells without KHYG-1 cells is expressed as 100%, and values for the other groups are expressed as percentages of the control. Data are the means ± SEM from six samples. * *p* < 0.05 and ** *p* < 0.01 when compared with LN cells in each group. E/T: the ratio of KHYG-1 cells to LN-stem cells or LN cells. (**C**) Co-culture of KHYG-1 cells with LN-stem cells (LNCaP CSC) or LN cells (LNCaP Con) was performed for 24 h, and CV staining was carried out. This result is representative of one of three independent experiments. E/T: the ratio of KHYG-1 cells to LN-stem cells or LN cells. Con: control (without KHYG-1 cells). Magnification ×20.

As KHYG-1 cells have been shown to possess strong cytotoxicity against cancer cells [15], we evaluated the expression patterns of cytotoxicity-related activating receptors on the surfaces of the NK cells. As shown in Figure 1A, the KHYG-1 cells expressed the activating receptors NKG2D, NKp30, and NKp46 on their surfaces. Next, we compared the sensitivity of LN-stem cells and LN cells to NK cell-mediated cytotoxicity. LN-stem cells showed a significantly greater sensitivity than LN cells to KHYG-1 cell-mediated cytotoxicity after co-culture for 24–72 h (Figure 1B). Similarly, CV staining clearly showed that KHYG-1 cell-mediated cytotoxicity against LN-stem cells was much higher than that against LN cells (Figure 1C).

### 3.2. Relationship between KHYG-1 Cell-Mediated Cytotoxicity against LN-Stem Cells and Activation Receptor NKG2D and Its Ligands

**Figure 2:**
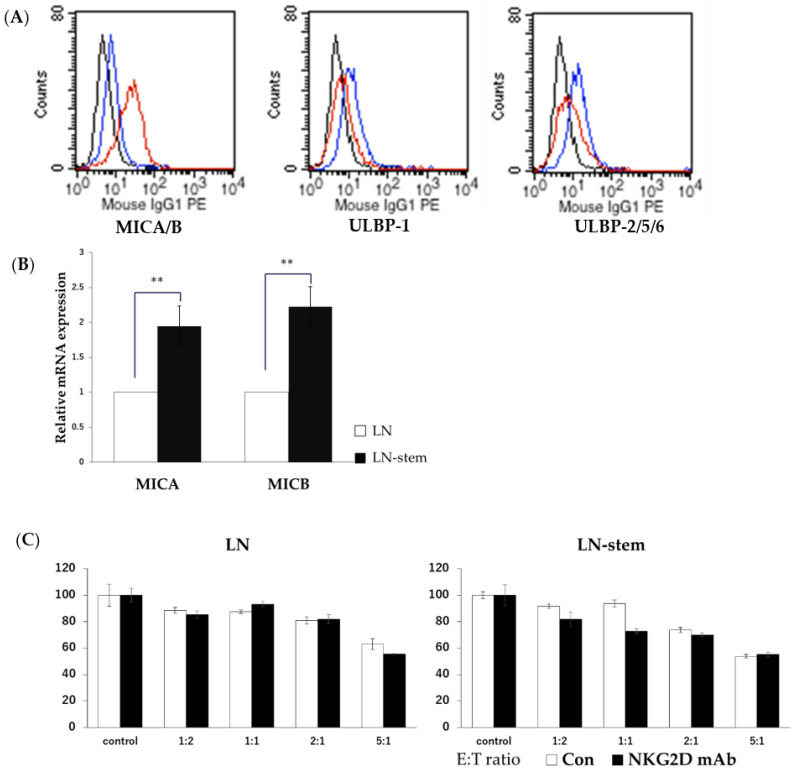
Relationship between NKG2D-MICA/B signaling and KHYG-1 cell-induced cytotoxicity against LN-stem cells. (**A**) Comparison of cell surface expression levels on NKG2D ligands (MICA/B, ULBP-1, and ULBP-2/5/6) between LN-stem cells and LN cells using FACS. Black, blue, and red lines indicate the matched isotype control, LN cells, and LN-stem cells, respectively. Values in the blue and red boxes indicate MICA/B-positive cell percentages for LN cells and LN-stem cells, respectively. (**B**) mRNA levels of MICA and MICB in LN cells and LN-stem cells. Each column indicates the mean, and vertical lines indicate SEM (n = 3). Values for LN cells are expressed as 1, and values for LN-stem cells are expressed as ratios relative to LN cells. ** *p* < 0.01 when compared with LN cells. (**C**) The effect of anti-NKG2D antibodies (treatment concentration 10 μg/mL) on KHYG-1-induced cytotoxicity against LN-stem cells or LN cells when co-cultured for 72 h. Cell viability was determined by the WST-8 assay. Values for the control are expressed as 100%, and values for the other groups are expressed as percentages of the control. Data are means ± SEM from six samples. E/T: the ratio of KHYG-1 cells to LN-stem cells or LN cells.

A previous study demonstrated that the main activation receptor on the surfaces of KHYG-1 cells is NKG2D [15], and, as shown in Figure 1A, we also observed the clear expression of NKG2D on the surfaces of the KHYG-1 cells. We then sought to establish which activation receptor-bound ligands are likely to be expressed on the surfaces of LN-stem cells. As shown in Figure 2A, FACS analysis showed that, of the NKG2D-bound ligands, MICA/B ligands were present at much higher levels in LN-stem cells than in LN cells (MICA/B-positive cells: LN-stem cells, 59.7%; LN cells, 1.7%). On the other hand, two other ligands bound to NKG2D, ULBP-1 and ULBP-2/5/6, had similar expression levels in LN-stem cells and LN cells (Figure 2A). In agreement with this result, the mRNA levels of MICA and MICB in LN-stem cells were significantly higher than in LN cells (Figure 2B). However, neutralization of the NKG2D function by the anti-NKG2D antibody treatment did not affect KHYG-1-dependent cytotoxicity against LN-stem cells or LN cells (Figure 2C).

### 3.3. Relationship between KHYG-1 Cell-Mediated Cytotoxicity against LN-Stem Cells and the Death Receptor Pathway

**Figure 3:**
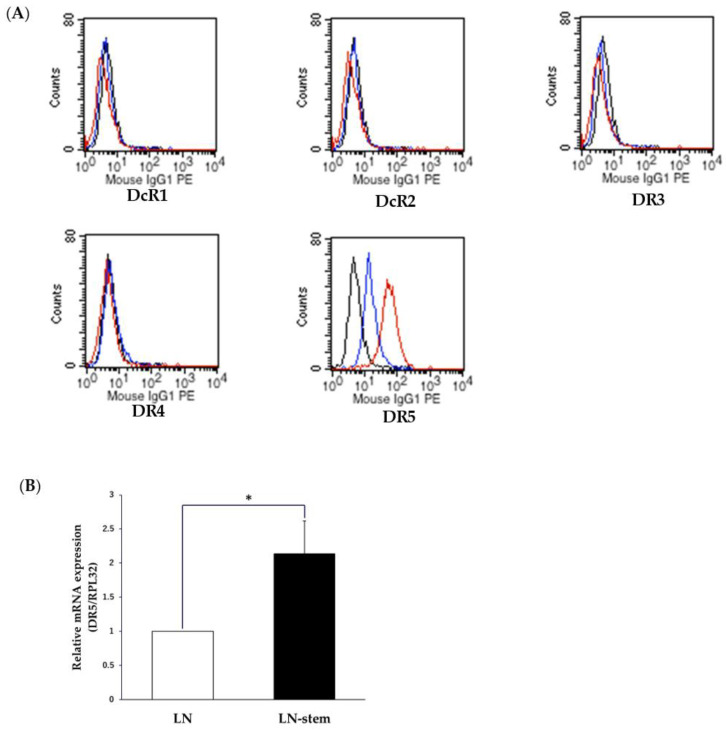

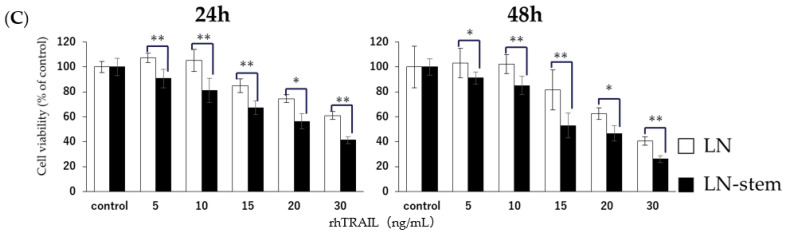
Relationship between TRAIL-DR5 signaling and KHYG-1 cell-induced cytotoxicity against LN-stem cells. (**A**) Comparison of cell-surface expression levels on TRAIL-related receptors (DcR1, DcR2, DR3, DR4, and DR5) between LN-stem cells and LN cells by FACS. Black line, blue line, and red line indicate matched isotype control, LN cells, and LN-stem cells, respectively. Values in the blue and red boxes indicate DR5-positive cell percentages for LN cells and LN-stem cells, respectively. (**B**) mRNA levels of DR5 in LN cells and LN-stem cells. Each column indicates the mean, and vertical lines indicate SEM (n = 3). Values for LN cells are expressed as 1, and values for LN-stem cells are expressed as ratios relative to LN cells. * *p* < 0.05 when compared with LN cells. (**C**) The effect of rhTRAIL on the viability of LN-stem cells and LN cells when co-cultured for 72 h. Cell viability was determined by the WST-8 assay. Values for the control are expressed as 100%, and values for the other groups are expressed as percentages of the control. Data are the means ± SEM from six samples. * *p* < 0.05 and ** *p* < 0.01 when compared with LN cells for each treatment dose.

CD133-positive human liver cancer stem-like cells have high expression levels of DR5 and are sensitive to TRAIL treatment [16]. Based on this, we estimated the expression patterns of five death receptors and decoy receptors related to TRAIL found on the surfaces of LN-stem cells and LN cells. FACS analysis indicated that the percentages of DR5-positive cells in LN-stem cells and LN cells were 97.6% and 19.5%, respectively (Figure 3A). This observation was supported by the significantly higher levels of DR5 mRNA expression in LN-stem cells than in LN cells (Figure 3B). However, there were no differences in the expression levels of other death and decoy receptors (DR3, DR4, and DcR1/2) on the surfaces of LN-stem cells and LN cells (Figure 3A). Furthermore, rhTRAIL treatment for 24–48 h significantly inhibited the viability of LN-stem cells in a dose-dependent manner when compared with LN cells (Figure 3C), suggesting that KHYG-1 may preferentially induce cytotoxicity against LN-stem cells via the TRAIL/DR5 death receptor pathway.

## 4. Discussion

Cancer stem cells constitute a small subpopulation of cancer cells capable of self-renewal and unlimited replication, which initiates tumor formation [17]. Cancer stem cells are also the primary causes of tumor resistance to conventional treatments and may survive initial therapies, leading to the recurrence, progression, and metastasis of prostate and several other cancer types after therapy [18,19,20,21]. Thus, it has been hypothesized that complete regulation of the development of prostate cancer stem-like cells may lead to the establishment of effective preventive measures against prostate cancer. Of previously reported methods of cancer stem cell regulation, we focused on the NK cell-dependent eradication of cancer stem cells because it has been reported that NK cells can specifically target cancer stem-like cells and inhibit the occurrence of cancer [22,23]. This study investigated whether NK cells can preferentially target prostate cancer stem-like cells and effectively induce cytotoxicity in these cells.

A previous study has clearly showed that the potent cytotoxic activity of KHYG-1 cells depends on the degranulation pathway being regulated by perforin and granzyme [15]. In this study, we also observed that a main activation receptor of NK cells, NKG2D, is essential for initiating the degranulation pathway. Additionally, with respect to the expression of NKG2D ligands, it is known that the induction of such ligands within cells can be caused by factors that stress the genome, such as phenotypes of cancer stemness [24], infection, and carcinogenesis [25]. Through the interaction of NKG2D and its ligands, the receptor can act as a main recognition receptor to sense and eliminate transformed and infected cells [24]. In this study, we showed that the ratio of MICA/B- (NKG2D ligands) positive cells and associated mRNA levels was much higher in LN-stem cells than in LN cells. These data suggest that KHYG-1 cells preferentially target LN-stem cells via the interaction of NKG2D and MICA/B, inducing cytotoxicity. However, anti-NKG2D antibody treatment did not attenuate KHYG-1 cell-mediated cytotoxicity against LN-stem cells, indicating that the degranulation pathway regulated by NKG2D-MICA/B is not involved in cytotoxicity. This observation is discordant with the observation of higher levels of MICA/B in LN-stem cells, and we are currently unable to explain this discrepancy. However, it has been reported that MICA/B proteins in breast cancer stem-like cells easily detach from cell surfaces [26], so our data may be explained by the detachment of these ligands from the surfaces of LN-stem cells. In order to resolve these conflicting data, further studies will be needed.

In addition to the degranulation pathway, the death receptor pathway with TRAIL as the death receptor ligand is known to be one of the NK cell-mediated cytotoxic pathways, and this death receptor pathway is representative of extrinsic apoptotic pathways [27]. After TRAIL binds to death receptors DR4/DR5, downstream molecules are activated, which induces apoptosis via the activation of the caspase cascade [28]. In this study, LN-stem cells were shown to have elevated DR5 mRNA and cell-surface protein levels compared with LN cells, which is in agreement with a previous report that found DR5 expression in liver cancer stem-like cells to be high [16]. Our study also shows that other TRAIL receptors, DR3 and DR4, and its decoy receptor, DcR1/2, are not expressed on the surfaces of LN-stem cells, so we speculate that KHYG-1 cells may preferentially induce cytotoxicity in LN-stem cells via the activation of the TRAIL/DR5 death receptor pathway. This speculation is supported by our observation that rhTRAIL cytotoxicity was more potent against LN-stem cells than LN cells. Furthermore, it seems to be possible that KHYG1-induced cytotoxicity against LN-stem cells mainly depends on apoptosis based on previous reports [29,30] and our preliminary observations. One study suggested that the expression and function of caspase-8 are central to TRAIL-mediated cell death, and that the activation of caspase-8 initiates a caspase cascade, ultimately leading to apoptosis [29]; the other study demonstrated that NK cell-induced activation of TRAIL/caspase-8 signaling is clearly associated with apoptosis, showing typically apoptotic features such as cell condensation and membrane blebbing [30]. Additionally, we observed that caspase-8 activation was induced in co-culture of KHYG-1 cells with LN-stem cells in our preliminary experiment.

CD73 has been implicated as an important factor in the induction of the high expression of DR5 in LN-stem cells. In a preliminary experiment, we observed the expression of CD73 in LN-stem cells to be higher than in LN cells. CD73 acts as an immune-suppressor by stimulating the expression of DR5 via AMP-activated protein kinase, resulting in elevated immune-suppressive adenosine production in cancer stem cells and subsequent inhibition of the mammalian target of rapamycin [31,32,33]. Thus, it seems plausible that the high expression of CD73 in LN-stem cells leads to the up-regulation of DR5. If the findings in this study can be shown to be true for human NK cells, the targeted eradication of prostate cancer stem cells by NK cells may become a feasible preventive strategy for prostate cancer.

## Figures and Tables

**Table 1:** List of PCR primers.

Gene	Primer	Sequence (5′–3′)
NKG2D	Forward primer	TGAGAGTAAAAACTGGTATGAGAGCCA
	Reverse primer	TGCATGCAGATGTATGTATTTGGAG
MICA	Forward primer	AGACTTGACAGGGAACGGAAAG
	Reverse primer	TCCAGGTTTTGGGAGAGGAA
MICB	Forward primer	ACCTTGGCTATGAACGTCACA
	Reverse primer	CCCTCTGAGACCTCGC
DR5	Forward primer	GTCGTTGTGAGCTTCTGTCC
	Reverse primer	GCCTCTCCCTGTTCTCTCTC
RPL32	Forward primer	AACCCTGTTGTCAATGCCTC
	Reverse primer	CATCTCCTTCTCGGCATCA

## Data Availability

All relevant data are within the paper.
